# Long-Term Mutual Training for the CYBATHLON BCI Race With a Tetraplegic Pilot: A Case Study on Inter-Session Transfer and Intra-Session Adaptation

**DOI:** 10.3389/fnhum.2021.635777

**Published:** 2021-02-26

**Authors:** Lea Hehenberger, Reinmar J. Kobler, Catarina Lopes-Dias, Nitikorn Srisrisawang, Peter Tumfart, John B. Uroko, Paul R. Torke, Gernot R. Müller-Putz

**Affiliations:** ^1^Institute of Neural Engineering, Graz University of Technology, Graz, Austria; ^2^Graz BCI Racing Team Mirage 91, Graz University of Technology, Graz, Austria; ^3^Information Integration and Neuroscience Team, RIKEN Advanced Intelligence Project, Kyoto, Japan; ^4^BioTechMed Graz, Graz, Austria

**Keywords:** brain-computer interface, spinal cord injury, CYBATHLON, mental imagery, inter-session transfer learning, long-term training, intra-session adaptation, rehabilitation

## Abstract

CYBATHLON is an international championship where people with severe physical disabilities compete with the aid of state-of-the-art assistive technology. In one of the disciplines, the BCI Race, tetraplegic pilots compete in a computer game race by controlling an avatar with a brain-computer interface (BCI). This competition offers a perfect opportunity for BCI researchers to study long-term training effects in potential end-users, and to evaluate BCI performance in a realistic environment. In this work, we describe the BCI system designed by the team Mirage91 for participation in the CYBATHLON BCI Series 2019, as well as in the CYBATHLON 2020 Global Edition. Furthermore, we present the BCI’s interface with the game and the main methodological strategies, along with a detailed evaluation of its performance over the course of the training period, which lasted 14 months. The developed system was a 4-class BCI relying on task-specific modulations of brain rhythms. We implemented inter-session transfer learning to reduce calibration time, and to reinforce the stability of the brain patterns. Additionally, in order to compensate for potential intra-session shifts in the features’ distribution, normalization parameters were continuously adapted in an unsupervised fashion. Across the aforementioned 14 months, we recorded 26 game-based training sessions. Between the first eight sessions, and the final eight sessions leading up to the CYBATHLON 2020 Global Edition, the runtimes significantly improved from 255 ± 23 s (mean ± std) to 225 ± 22 s, respectively. Moreover, we observed a significant increase in the classifier’s accuracy from 46 to 53%, driven by more distinguishable brain patterns. Compared to conventional single session, non-adaptive BCIs, the inter-session transfer learning and unsupervised intra-session adaptation techniques significantly improved the performance. This long-term study demonstrates that regular training helped the pilot to significantly increase the distance between task-specific patterns, which resulted in an improvement of performance, both with respect to class separability in the calibration data, and with respect to the game. Furthermore, it shows that our methodological approaches were beneficial in transferring the performance across sessions, and most importantly to the CYBATHLON competitions.

## Introduction

Learning to control an application with a brain-computer interface (BCI) is a tale of two learners (Pfurtscheller and Neuper, 2001; Perdikis et al., 2020). In the case of oscillatory BCIs that detect power modulations in brain rhythms associated with distinct mental tasks (Wolpaw et al., 2002), ideally both the brain/user and the computer learn. Given a set or stream of data, machine learning is applied to detect and track user-specific patterns associated with mental tasks. The user receives feedback about whether the computer has detected an intended mental task, and uses this feedback to strengthen and consolidate the brain patterns. Two factors facilitate learning in this situation. First, the user requires knowledge about how s/he should perform the mental tasks (Neuper et al., 2005; Friedrich et al., 2012). Second, the computer requires a reasonable initial model of the user’s patterns. Initial models are typically obtained from user- and session-specific calibration data (Pfurtscheller et al., 1999; Guger et al., 2001; Pfurtscheller and Neuper, 2001), previous sessions (Perdikis et al., 2018), or sometimes other users (Kobler and Scherer, 2016; Zanini et al., 2018). As highlighted in Perdikis et al. (2020), current research trends are clearly biased toward evaluating BCI operation within one or few sessions, while there is relatively little BCI literature on long-term user training (Pfurtscheller et al., 2000; Neuper et al., 2003; Wolpaw and McFarland, 2004; Kübler et al., 2005; Saeedi et al., 2017; Müller-Putz et al., 2019). Nevertheless, long-term user training with BCIs can also be applied in the context of rehabilitation of stroke patients (Ang et al., 2009; Mrachacz-Kersting et al., 2016; Mane et al., 2020).

CYBATHLON provides an ideal platform to study long-term training effects in end-users. It was founded as a championship to connect people with physical disabilities and technology providers (Riener, 2016). In six disciplines, end-users, called pilots, use assistive technology to compete by completing everyday tasks as fast and accurately as possible. The championship takes place every 4 years, giving the teams time to develop and customize assistive devices for their pilots. In one discipline, called BCI Race, tetraplegic pilots have to control an avatar along a virtual race track (Novak et al., 2017). The discipline requires three discrete input commands associated to specific sections along the race track, and a non-command state. Correct commands are rewarded by an acceleration of the avatar, while wrong commands result in deceleration. The pilot’s task is to control the avatar by voluntarily modulating his/her brain activity without relying on external stimuli. A customized BCI translates the modulations into discrete commands which ideally match the user’s intention and make the vehicle pass the track quickly. In CYBATHLON, the focus is on non-invasive technologies that are portable and affordable. Electroencephalography (EEG) fulfills these requirements, rendering it the single functional brain imaging technique that was used in the last championship in 2016 (Novak et al., 2017). Since external stimuli are prohibited, the teams relied on modulations of oscillatory brain activity during specific mental tasks—primarily on modulations of sensorimotor rhythms (SMRs) during motor imagery/attempt (Novak et al., 2017).

Following the CYBATHLON 2016, a survey revealed that the training strategies and protocols varied considerably across teams (Perdikis et al., 2018). Some teams started training more than a year before the competition, while others started a month before the competition. The number of training sessions ranged from 9 to 35. For this competition, our team employed a multi-stage BCI calibration procedure that started approximately 12 months before the competition with one session with the pilot per month in the early stages, and an intensification before the competition (Statthaler et al., 2017). While other teams used as few as two mental imagery tasks and a temporal coding (Perdikis et al., 2018), our strategy was to implement the three commands and the non-command state with four distinct mental tasks. After our pilot learned to produce four patterns that could be reasonably well-discriminated against each other, we started training with the game. Within eight training sessions, our pilot was able to improve the average runtime from 178 to 143 s. After the final competition specifications with a shorter track length were released, the average runtime converged to 120 s. In the competition, we observed a substantial performance drop; our system achieved a runtime of 196 s in the qualification run. The *Brain Tweakers* team observed a similar drop in one of their two pilots (Perdikis et al., 2018). While this pilot achieved the fastest runtime in the qualification runs (90 s), in the finals, his runtime was 190 s.

In our case, several factors introduced non-stationarities to the EEG signals which lead to a shift in the feature distribution, invalidating the fitted classifier (Statthaler et al., 2017). These non-stationarities or variations could have physical or mental origin. Physical variations include changes in the electrode scalp interface within or across sessions. Mental variations are caused by non-task relevant brain activity due to different mental conditions (Ahn and Jun, 2015). For example, the arousal level in a lab and a stadium full of spectators might differ. A common strategy to overcome these intra-subject variations is to extract invariant features (Samek et al., 2014; Morioka et al., 2015) or compensate changes in the overall feature distribution (Vidaurre et al., 2011a). In this work, we applied an adaptive feature normalization approach to fit and evaluate a four class BCI to a new pilot in a longitudinal study over the course of 14 months. The duration of the study covers the preparation period for the participation in the CYBATHLON BCI Series 2019, a single-discipline spin-off event of the CYBATHLON, as well as the CYBATHLON 2020 Global Edition.

## Materials and Methods

### Pilot

The pilot was 33 years old at the time of the CYBATHLON 2020 Global Edition. He was recruited by a neurologist at AUVA Rehabilitation Clinic Tobelbad, who proposed him to the team. He has had a spinal cord injury (SCI) since an accident in 2007. His neurological level of injury (NLI) is C5 (AIS B). He is able to flex the elbow in full range (grade 5) but extend only with a grade of 2. He can extend his left wrist with grade 5 and the right one with grade 4. He has neither finger flexion, nor extension (grade 0). His shoulder movement is mainly intact, so he can drive his supported (e-motion Alber GmBH) wheelchair by himself.

### Training Protocol

**FIGURE 1:**
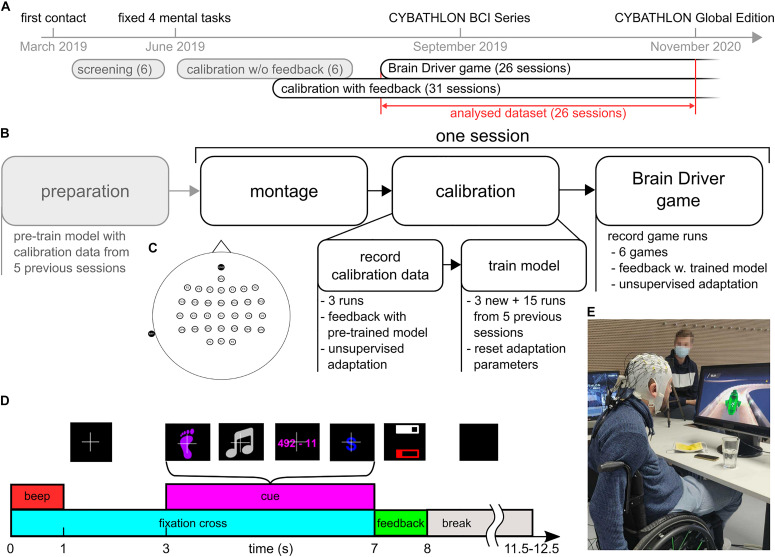
Overview of the training protocol. **(A)** Timeline from the first contact with the pilot until the CYBATHLON 2020 Global Edition. This study considers 26 sessions that contained calibration with feedback and game data. **(B)** Protocol of the 26 sessions. Before the session, classification and artifact detection models were pre-trained using the calibration data from the five previous sessions (15 runs). After mounting the cap and electrodes, three calibration runs with 40 trials each were recorded. During these runs, the pilot received feedback based on the pre-trained models. The calibration data were subsequently used to retrain the models. The retrained models were used to play the Brain Driver game. **(C)** Electrode layout. Thirty-two channels were placed at frontal, central and parietal locations. Reference and ground were placed at the left mastoid and Fpz. **(D)** Sequence of events during a calibration trial with feedback. The pictures above the events depict the associated screen content presented to the pilot. At the beginning of each trial, a fixation cross appeared, and an audible beep was played. After a fixation period of 3 s, a visual cue informed the pilot of the current mental task. The pilot performed the respective task while the cue was on screen. After the cue period, the pilot received feedback about the target class, summarized as the average target class probability during the last 3 s of the cue period. The probability was presented as a white bar within a white frame. If the target class’ average probability was either below 25% or not the largest, the frame was empty. The artifact probability during the same period was summarized in a similar fashion (red bar and frame). Breaks between trials lasted between 3.5 and 4.5 s. **(E)** Pilot with EEG setup, playing the BrainDriver game.

Figure 1A presents an overview of the training timeline. During the initial 4 months of working with the pilot, we performed screening sessions where we tested several class combinations out of a pool of eight classes. The following mental tasks were screened: feet motor imagery, hand motor imagery, singing, mental subtraction, mental rotation, spatial navigation, word association, and face recall. Detailed task descriptions can be found in Friedrich et al. (2012).

For the screening procedure as well as to collect calibration data in the subsequent regular training sessions, we used a modified version of the Graz BCI motor imagery paradigm (Pfurtscheller and Neuper, 2001) with discrete visual feedback, as shown in Figure 1D. Each trial started with a white fixation cross on a black screen and with an acoustic beep in order to attract the attention of the pilot. After 3 s, a visual cue representing one of the mental tasks appeared on the screen. The pilot was instructed to perform the respective mental task for as long as the cue was on the screen. The cue period lasted for 4 s. Between trials, there was a short break, which lasted between 3.5 and 4.5 s, where the screen was black.

In the screening sessions, EEG signals during the cue period were evaluated offline, and no feedback was provided. This strategy to collect calibration data is identified as “calibration w/o feedback” in Figure 1A. At the end of the screening period, we selected the tasks feet motor imagery, singing, mental subtraction, and word association. This decision was based on the existence of distinguishable brain patterns in event-related (de)synchronization (ERDS) maps (Pfurtscheller and Lopes da Silva, 1999; Graimann et al., 2002), and on the preferences of the pilot. In the subsequent training sessions, we incorporated discrete visual feedback into the collection of calibration data (“calibration with feedback”). To this end, a classification model was trained prior to each session, based on EEG data from the five preceding sessions, and used to provide online feedback during the calibration runs. During these calibration runs with feedback, following the cue period, two horizontal feedback bars were displayed for 1 s: a white feedback bar reflecting the class probability in case of a correctly classified trial, and a red feedback bar indicating the probability of the presence of an artifact during the trial. In this study, we consider the main training period (26 sessions). The typical protocol for these sessions is illustrated in Figure 1B. In each session, we recorded three calibration runs with feedback, each comprising 40 trials: 10 trials per class in a pseudorandom order. Afterward, the classification model was updated, and the pilot completed 6 ± 1.3 (mean ± SD) game runs with the BrainDriver game. A typical training session lasted between 120 and 150 min, which included between 10 and 30 min of montage and setup on-site, calibration runs, retraining of the classification model, game runs, and breaks. The net recording time was approximately 60 min, and the number and duration of breaks were dictated by the pilot.

### BrainDriver Game

The BrainDriver program simulates a race in which pilots have to steer a vehicle along a race track (Figure 1E). The race track consists of the following four sections: straight, left curve, right curve, and dark area. The sections are equally distributed over the entire distance of the track. The game accepts three possible commands, corresponding to three in-game actions, i.e., left turn, right turn, and headlights. Each of these commands is accepted as a correct command on the appropriate sections, i.e., left and right turns on left and right curves, respectively, and headlights on dark areas. On straight sections, any command is incorrect. All commands to the vehicle are issued via the pilot’s BCI system. The vehicle remains in motion throughout the whole race, whereas the speed is modulated by correct and incorrect commands.

Due to the four specified sections of the racetrack, we decided to employ a 4-class BCI system, and to assign one class to each of the three commands, and the fourth class to the non-command state. Specifically, the classes feet, subtraction, and word, were associated with the game commands, “right,” “left,” and “headlights,” respectively, whereas the class singing was associated with the non-command state. Furthermore, the probability of artifact contamination is supplied by the artifact detector.

Online, the classification model yields a set of four probabilities, corresponding to the four classes, at each time point. Following a smoothing of the class probabilities via a moving-average filter with a window length of 1.4 s, the class with the maximal probability is pre-selected, and several conditions are assessed to determine whether the respective command should be triggered. These conditions serve to regulate the sending of commands to the game, in order to pre-empt issues arising due to a classifier bias, or fluctuations in the class probabilities. Furthermore, no commands may be sent if the artifact probability exceeds 0.5, lest the game be controlled by artifacts.

**FIGURE 2:**
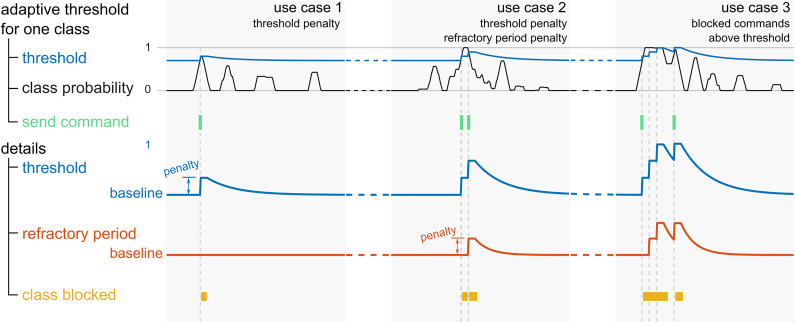
Illustration of the adaptive threshold and refractory period of an exemplary class in three use cases. The top row depicts the class probability (black traces), the corresponding adaptive threshold (blue traces), and the time points where the corresponding command is triggered (green). In the bottom rows, a zoomed in view of the threshold, and the similarly adaptive refractory period (orange) are shown. At the bottom, the periods where the class is blocked are marked in yellow.

Principally, the probability of the pre-selected class must exceed a certain threshold. This threshold is initialized at an empirically selected baseline value, and adapted throughout the runtime, for each class separately. More specifically, each time the class in question is pre-selected, and exceeds the threshold at that time point, the respective threshold is raised. The raising of the threshold is followed by a refractory period, and a consequent exponential decay back to the baseline value (use case 1 in Figure 2). If the threshold of the pre-selected class is higher than a defined value above the baseline threshold before it is being raised, the refractory period of the respective class is extended (use case 2 in Figure 2). The duration of the refractory period slowly reverts back to its baseline value with an exponential decay. As long as a refractory period is active, no command can be sent to the game, and the thresholds as well as the durations of the refractory periods of all four classes are held constant. As an additional measure to mitigate the effect of an extreme classifier bias toward one class, a command may not be sent if the class probability is higher than 0.99, and the same class has just been triggered (use case 3 in Figure 2).

### Equipment and Layout

In all sessions, the pilot’s EEG activity was recorded at 500 Hz using a LiveAmp amplifier (BrainProducts GmbH, Germany) with 32 active electrodes. The electrodes were placed in a 10-10 layout over frontal, central and parietal areas, as depicted in Figure 1C. The reference electrode was placed on the left mastoid and the ground was located at Fpz.

We used the labstreaminglayer (LSL) protocol^1^ to synchronize the EEG signals with the events triggered by the paradigm and the BCI system. A custom BCI system and analysis scripts were developed using Matlab (2015b, Matworks Inc., United States) and the EEGLAB toolbox (Delorme and Makeig, 2004). The calibration paradigm was based on the Psychtoolbox for Matlab (Brainard, 1997; Pelli, 1997).

### Pipeline

**FIGURE 3:**
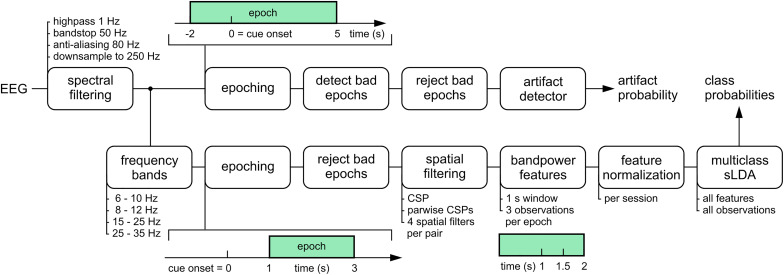
Offline processing pipeline used to fit the model parameters to calibration data. First, the raw EEG signals were filtered and sent through two processing branches. In the upper branch, bad epochs were detected and rejected. The remaining epochs were used to train the artifact detection model. In the lower branch, the parameters of the FBCSP + sLDA classification model were fitted. Four CSP filters were trained for each of the four frequency bands and binary class combinations. After CSP filtering, log band power features were extracted and normalized per session. Finally, a multiclass sLDA filter was fitted to all features and observations to predict the probability for each of the four classes.

The signal processing pipeline used for fitting the classification model is depicted in Figure 3. In the first step, drifts and line noise were removed with a highpass filter (2nd order Butterworth filter, 1 Hz cutoff frequency) and a bandstop filter (2nd order Butterworth filter, 49 and 51 Hz cutoff frequencies). Then, an anti-aliasing lowpass filter was applied (8th order Butterworth filter, 80 Hz cutoff frequency). Afterwards, the EEG signals were downsampled to 250 Hz.

From this point on, the pipeline diverged into two different branches (Figure 3). The upper branch was used 2-fold. First, trials affected by artifacts were detected and rejected. Second, the artifact detection model was trained. The lower branch was used to fit the parameters of a 4-class filter bank common spatial patterns (FBCSP) + linear discriminant analysis (LDA) classification model (Ang et al., 2008).

In the upper branch, the preprocessed signals were segmented into epochs of [−2, 5] s with respect to the onset of the cue (*t* = 0 s). In the next step, outliers at the level of these epochs were detected (Delorme and Makeig, 2004) and marked for subsequent rejection. This resulted in 13 ± 4% (mean ± std) rejected epochs per session. Afterward, the artifact detection model parameters were fitted. The algorithm is based on the high-variance electrode artifact removal (HEAR) algorithm (Kobler et al., 2019).

In a nutshell, HEAR monitors the variance of each EEG channel and converts it into an artifact probability by comparing the time-varying variance at each time point with the variance of (artifact-free) calibration data; in this case, the remaining epochs after artifact rejection. It is therefore possible to detect high variance artifacts (e.g., blinks, muscle artifacts, pops, and drifts). As outlined in Kobler et al. (2019), the artifact probability can be used to attenuate single electrode artifacts via interpolating the affected channels activity based on the activity of neighboring channels. We did not use this feature in this study.

Since the artifact probability was used to block the BCI output, we decided to slowly adapt the reference variances per channel. Without adaptation, a change in the overall tension of the pilot would have resulted in a sustained blocking of the BCI output and potential disqualification. This adaptation method was identical to the adaptive feature normalization method, outlined in subsection intra-session adaptation.

In the lower branch, the activity of four frequency bands was extracted from the preprocessed EEG signals. The bands were selected according to class-specific effects in the screening data. The selected bands were partially overlapping and covered the alpha and beta bands. To extract signals in each frequency band, bandpass filters (3rd order Butterworth filters, the individual cutoff frequencies are listed in Figure 3) were applied. The filtered EEG signals were then segmented into epochs of [1, 3] s with respect to the cue. Next, the previously marked outlier epochs were rejected. The number of epochs per class was then adjusted to the number of epochs of the class that contained the maximum number of remaining epochs. The added epochs were randomly selected from the class’ epochs.

The equalized epochs were used to fit the parameters of common spatial pattern (CSP) spatial filters (Ramoser et al., 2000; Blankertz et al., 2008) for each of the four frequency bands (Ang et al., 2008). Within each frequency band, binary CSP filters were trained in a one-vs.-one classification scheme, yielding six binary combinations for the four-class problem. Analytical shrinkage regularization (Schäfer and Strimmer, 2005) was used to estimate the class-specific covariance matrices from the samples of all epochs within the training set. From all possible CSP filters, the filters associated to the two highest and lowest eigenvalues were maintained. These CSP filters were applied to the band-specific EEG signals. From the resulting signals, log band power features were extracted, by squaring the signals, applying a 1-s moving average filter and taking the base 10 logarithm. For each epoch and feature, we extracted three observations at 1.0, 1.5, and 2.0 s. This resulted in a total of 334 ± 72 (mean ± std) observations per session and 96 features = 4 (frequency bands) × 6 (binary class combinations) × 4 (CSP filters). Before the log bandpower features were used to fit the parameters of a shrinkage regularized LDA (sLDA) classifier (Blankertz et al., 2011), each feature was normalized with the session-specific mean and standard deviation. Normalizing the features within each session can compensate for potential shifts in the features’ distribution across sessions.

As outlined in Figure 1B, the parameters of the pre-trained model, used to provide feedback in the calibration runs, were fit to the data of five previous sessions. After the calibration data were recorded, the new data were included in the training set and the model was retrained. The retrained model was used during the BrainDriver games.

### Intra-Session Adaptation

To compensate for potential intra-session shifts in the features’ distribution, the pre-trained and retrained models were adapted during the calibration and game runs. Unlike adapting the parameters of the LDA model, as proposed in Vidaurre et al. (2011a, b), exponential moving average (EMA) filters were used to track the time-varying mean m_*t*_ and standard-deviation s_*t*_ of the (1 × *n*) dimensional feature vectors. At each time point *t*, the new observation x_*t*_ is used to update m_*i*_ and s_*t*_. Given the estimates of the previous time point *t-1*, the update rule for each component *i* is obtained by

(1) $$m_{t}^{\left(i\right)}=\left(1-\eta \right)m_{t-1}^{\left(i\right)}+\eta x_{t}^{\left(i\right)}$$

(2) st(i)=(1-η)st-1(i)2+η(xt(i)-mt(i))2

with a smoothing parameter η, which weights the contribution of the new observation to the normalization parameters.

We used the normalization parameters of the previous session as initial estimates (m_0_ and s_0_) to start feedback training with the calibration paradigm. After the model was retrained, the mean and standard deviation of the current session’s calibration data were used as initial estimates (m_0_ and s_0_) for the first game. During the remainder of the session, the model parameters were updated during game runs according to Eqs. (1 and 2), if the artifact probability of the current observation was lower than 0.5.

The smoothing parameter η was calculated as

(3) $$\eta =1-{\left(1-p\right)}^{\frac{1}{k}}$$

where *k* denotes the number of observations whose weights sum to a fraction *p* of the total EMA weights. We set *p* and *k* so that the samples within the last *k* = 10 min contained *p* = 90 % of the weights.

At each time-point *t*, the normalized feature vector ${\tilde{\text{x}}}_{t}$ was calculated as

(4) $${\tilde{\text{x}}}_{t}=\left({\text{x}}_{t}-{\text{m}}_{t}\right){\text{∑}}_{t}^{-\frac{1}{2}}\sum_{t}^{\frac{1}{2}}=\text{diag}\left({\text{s}}_{t}\right)=\left[\begin{array}{ccc} s_{t}^{\left(1\right)} & 0 & 0\\ 0 & \ddots & 0\\ 0 & 0 & s_{t}^{\left(n\right)} \end{array}\right]$$

where m_*t*_ and ${\text{∑}}_{t}^{\frac{1}{2}}$ denote the updated mean feature vector and the square root of the diagonal covariance matrix, respectively.

### Performance Evaluation

#### Patterns

The calibration data were used to identify whether the pilot’s patterns changed during the study. The patterns were extracted from channel-level log band power features for each class and session. In detail, the time-varying band power for each EEG channel was computed. Then, three observations per trial were extracted, yielding a total of 9,360 (=26 sessions × 3 runs/session × 4 classes/run × 10 trials/class × 3 observations/trial) observations for the 128 (=32 channels × 4 bands) features. After bad trials were rejected, 8628 observations (=92%) remained. Next, the observations per feature were divided by their average (all observations within a session) and log transformed. The resulting features expressed relative band power changes to the session’s average power in dB. The class- and session-specific patterns were then obtained via averaging the associated observations, resulting in 104 (=26 sessions × 4 classes) patterns.

Next, we quantified whether the discriminability of the patterns improved during the study. As metric, we computed ratios of the between- and within-class distances. The distances were computed by extracting two means per class *i* and session *s*, with the first mean μ_**1**_(*s*,*i*) summarizing the pattern of the first half of the observations and the second mean μ_**2**_(*s*,*i*) the last half. For each binary class combination (*i*,*j*), the within-class distance was then the sum of the Euclidean distances of the within class means

(5) $$\begin{array}{c} ||\Delta within\left(s,i,j\right)||=||\mu_{1}\left(s,i\right)-\\ \mu_{2}\left(s,i\right)|{|}_{2}+||\mu_{1}\left(s,j\right)-\mu_{2}\left(s,j\right)|{|}_{2} \end{array}$$

Conversely, the between-class distance was the sum of the distances between the patterns of different classes

(6) $$\begin{array}{c} ||\Delta between\left(s,i,j\right)||=||\mu_{1}\left(s,i\right)-\\ \mu_{2}\left(s,j\right)|{|}_{2}+||\mu_{1}\left(s,j\right)-\mu_{2}\left(s,i\right)|{|}_{2} \end{array}$$

This resulted in a total of 6 (=binary class combinations) between to within class distance ratios per session. For each ratio, we tested for significant trends across sessions and differences between the first eight and last eight sessions (for details see “Statistics” section).

To visualize the entire feature space, we applied t-distributed stochastic neighborhood encoding (t-SNE) (van der Maaten and Hinton, 2008). Specifically, t-SNE was used to reduce the 128-dimensional feature space to 2 dimensions with the goal to preserve the distances between the individual points without using information about the associated classes. The dimensionality reduction was applied to all 8,628 observations and 104 patterns simultaneously. Next, we estimated class specific distributions at the level of session groups (first 8, middle 10, last 8) in a non-parametric fashion. Using the subset of observations within the associated group and class, we then computed 2D histograms (25 ^∗^ 25 bins), applied smoothing (2D Gaussian kernel, σ = 2 bins) and normalized the histogram to obtain an empirical probability density function (PDF). The PDFs were used to visualize the dispersion of the group and class specific observations.

#### Inter-Session Transfer

To assess the effectiveness of the feature normalization with respect to inter-session transfer learning, we conducted offline analyses on the calibration data of 26 sessions. Each of these sessions had five preceding sessions that contained at least three calibration runs with feedback. To this end, we evaluated several models using specific selections of training and testing data. Table 1 contains an overview of the models and data splits. As a baseline method, we fitted the classification model to the data of a single session using a 5 × 10-fold cross validation (CV) scheme. Next, transfer models (without and with feature normalization) were trained on the data of the five chronologically preceding sessions, and tested on the respective target session. Note that the transfer model with normalization corresponded to the pre-trained model that was used to provide feedback during calibration runs. The combined models were fit to the combined training set of the single session and transfer models. The normalization parameters were calculated and applied for each session for the models with feature normalization. To identify significant differences in the test set accuracy between the models, two-sided, paired permutation (10,000 permutations) *t*-tests were computed for all binary model combinations (26 sessions, *df* = 25, 10 tests).

**TABLE 1:** Overview of the evaluated train test partitions and feature normalization methods.

Model	Train set	Test set	Cross validation	Normalization
Single session	9 CV folds	1 CV fold	5 × 10-fold	Yes
Transfer w/o norm	5 previous sessions	Target session	No	No
Transfer w norm				Yes
Combined w/o norm	5 previous sessions + 9 CV folds	1 CV fold	5 × 10-fold	No
Combined w norm				Yes

Additional tests were computed for each model to identify significant trends across sessions and differences between the first eight and last eight sessions (details see “Statistics” section).

#### Intra-Session Adaptation

The recorded EEG data of the BrainDriver game runs were used to evaluate the adaptive feature normalization approach. We conducted two simulations, denoted, with adaptation and without adaptation. In the first simulation (with adaptation), each new sample of the game runs updated the normalization parameters according to Eqs. (1 and 2). In the second simulation (w/o adaptation), the normalization parameters were not updated. Out of the 26 sessions, there was one session without recorded EEG data during the game runs, limiting this analysis to 25 sessions.

The processing pipeline of the simulations was similar to the online system. First, as outlined in Figure 3, the EEG signals were spectrally filtered. Then, the artifact detector was applied to compute the artifact probabilities for each time point. In parallel, the activity for each of the four frequency bands was extracted, the CSP filters applied and log bandpower features computed. In the simulation without adaptation, the features were normalized with the mean and standard deviation estimated from the session’s calibration data. In the simulation with adaptation, the parameters were adapted according to Eqs. (1 and 2). In both simulations, the class probabilities of the sLDA classifier were saved.

To estimate the effectiveness of the adaptation approach in compensating intra-session non-stationarities, the biases of both classification models (with and without adaptation) during Brain Driver games were used as a proxy metric. Non-stationarities typically result in shifts in the overall feature distribution (Vidaurre et al., 2011a), rendering the non-adaptive model suboptimal and resulting in biases toward specific classes. By design, the occurrence and length of the classes is equally distributed for one game. Consequently, a model that can track shifts in the feature distribution should select each class equally often; i.e., exhibit no bias.

To identify biases, the mean class probabilities for each game and session were computed and compared. Then, the difference to the probability of an unbiased classifier (25%) was computed. A two-sided, permutation (10,000 permutations), paired *t*-test was used to test for a difference in the biases between the adaptive and non-adaptive model across sessions (25 sessions, *df* = 24).

#### Statistics

This study’s primary focus was on training effects across sessions. Training effects were identified 2-fold. First at the session level, by computing Pearson correlation coefficients *r* between the session index, encoded as elapsed time since the first session, and each metric. Second at the session group level, two-sample *t* statistics were computed between the first eight and last eight sessions. In either case, non-parametric permutation (10,000 permutations) tests (Nichols and Holmes, 2002; Maris and Oostenveld, 2007) were used. The considered metrics were the game runtimes, the pattern distance ratios and the test set accuracies of the evaluated inter-session transfer models, resulting in a total of 24 (=2 tests for 1 runtime, 6 ratios, 5 models) tests.

To test whether the effects in the metrics were related, we conducted three additional tests. At the session level, we tested for significant correlations between the runtime, the accuracy of the combined model with normalization, and the average (all binary comparisons) pattern distance ratio.

A total of 38 = 24 (metric × session) + 3 (metric × metric) + 10 (model × model) + 1 (intra-session adaptation) tests were computed. We corrected the significance level α = 0.05 of the individual tests to account for multiple comparisons by controlling the false discovery rate (FDR) according to (Benjamini and Hochberg, 1995).

## Results

For the evaluation of our results, we considered 26 training sessions that took place during the period between 03.09.2019 and 12.11.2020. We present the evolution of performance metrics at the level of three session groups (first 8 sessions, middle 10 sessions, and last 8 sessions). The first eight sessions correspond to the period between 03.09.2019 and 09.12.2019. The middle 10 sessions correspond to the period between 06.02.2020 and 07.09.2020. The last eight sessions correspond to the period between 05.10.2020 and 12.11.2020.

### Patterns

**FIGURE 4:**
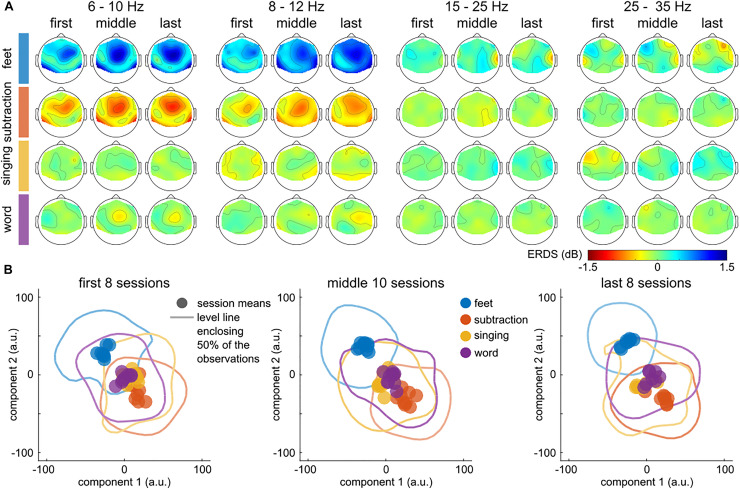
Calibration data. Evolution of class-specific patterns across training sessions. **(A)** Topographic distribution of the class-specific (rows) pattern across the first 8, middle 10 and last 8 sessions (columns). The evolution of the patterns is plotted separately for the four frequency bands (6–10 Hz, 8–12 Hz, 15–25 Hz, and 25–35 Hz). Each topographic plot summarizes the ratio between the class-specific and the grand average (=all classes) power per session. The group (first, middle, last sessions) level averages were computed in log space (dB). **(B)** Distribution of the session- and class-specific patterns within each group. t-SNE was used to compress the 128-dimensional feature space to 2 dimensions and maintain the distances between the individual observations. Each point summarizes the class-specific average of the observations within a session. The level lines summarize the dispersion of the observations within each class and group of sessions. Each line encloses an area that contains 50% of the estimated probability densities. Plots with multiple level lines are displayed in Supplementary Figure 1. The color indicates the associated class. To emphasize the evolution across session groups, they are plotted in three panels.

Figure 4 summarizes the evolution of the patterns over time, for the three session groups detailed above. Figure 4A depicts the evolution of the grand average log band power features over time. The frequency bands depicted are the ones used to train the classifier. This figure shows an enhancement of the patterns corresponding to the classes feet and subtraction, in the frequency bands [6, 10] Hz and [8, 12] Hz.

Figure 4B depicts the distribution of the session- and class-specific patterns in three panels, one for each group of sessions. A level line, enclosing 50 % of the estimated probability densities, summarizes the dispersion from the patterns. An increase in distance between the classes feet and subtraction over the training period indicates that the patterns became more distinguishable, which is also corroborated by the results of Figure 4A. In the last eight sessions, two clusters associated with feet and subtraction were separated from singing and word. The effect was significant for the (feet, subtraction) pattern distance ratio in terms of correlation (*r* = 0.47) and *t*-statistic (*t* = 3.32). Supplementary Figure 2 and Supplementary Table 1 summarize the results of all tests. Additional significant differences were observed for the (feet, word; *r* = 0.59, *t* = 3.99) and (subtraction, word; *r* = 0.47, *t* = 3.00) tuples. No significant effect was observed for any tuple that included the singing class.

### Evolution of Game Performance

**FIGURE 5:**
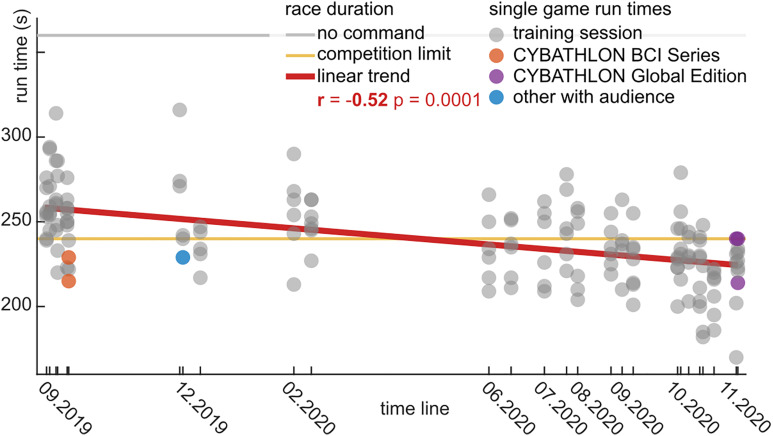
BrainDriver game runtimes for the 15 month training period in chronological order. Each dot represents the runtime during a game for training sessions (gray), the CYBATHLON BCI Series (orange), and Global Edition (violet) competitions and other sessions in front of an audience (blue). During the competitions the maximal runtime was limited to 240 s (yellow line). A horizontal gray line indicates the game duration in case no command is sent. A red regression line summarizes the linear trend across the training sessions.

The runtimes with the BrainDriver game are depicted in Figure 5. We observed a significant linear trend (*r* = −0.52, *p* = 0.0001), which highlights the decrease in runtimes over the training period. The slope of the linear trend was −2.4 s/month.

In the first eight sessions, the average runtime was 255 ± 23.1 s (mean ± std). This average decreased to 239 ± 20.4 s (mean ± std) in the middle 10 sessions, and further to 225 ± 21.7 s (mean ± std) in the final eight sessions. The difference between the first and last sessions was significant (*t* = −6.72, *p* = 0.0001). The last two sessions were performed with the competition setup, where races timed out at 240 s. In the cases where a time-out occurred, we extrapolated an estimated runtime based on the end position on the track.

### Inter-Session Transfer

**FIGURE 6:**
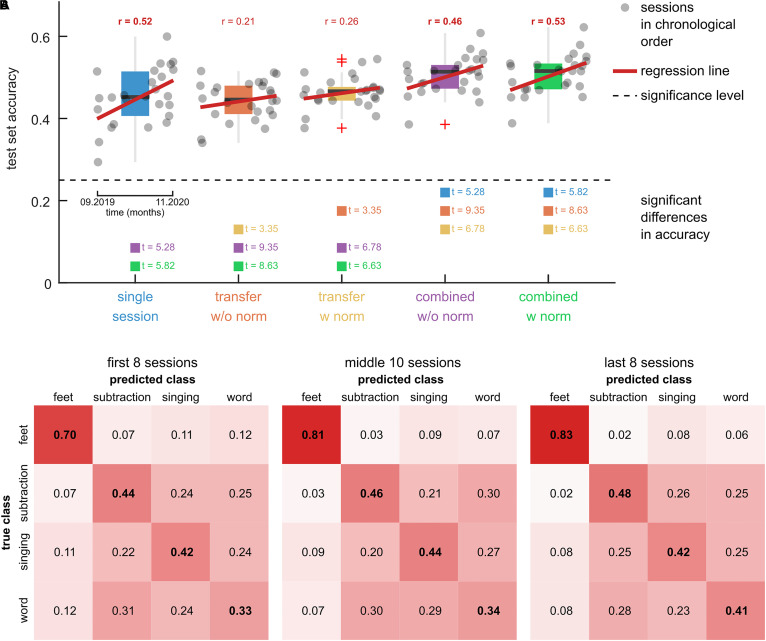
Comparison of model training approaches. **(A)** Boxplots of the test set accuracies of 5 models on the calibration data of 26 training sessions. The single session model was trained and tested on data of 1 out of the 26 target sessions using a CV scheme. The transfer models were trained on five previous sessions and tested on the data of the target session. Two variants of the transfer models were tested, namely, without feature normalization (w/o norm) and with feature normalization (w norm). The combined models were fit to the combined training dataset of the single session and transfer models. The dots summarize the test set accuracies of the 26 target sessions in chronological order. A black dashed horizontal line indicates the significance level. Red regression lines and correlation coefficients indicate trends over time. Significant trends are indicated (bold correlation coefficient). Model pairs with significant differences in the mean accuracy are highlighted and the associated t-statistic is reported. **(B)** Confusion matrices for the combined model with feature normalization. Three groups of sessions, namely the first 8 sessions, the second 10 sessions, and the last 8 sessions are summarized in separate panels. The elements in the main diagonal summarize the within-class accuracy. The rows sum to 1.

To evaluate the inter-session transfer, we computed the overall accuracies for the five tested models on the calibration data. The results are summarized in Figure 6. Figure 6A shows a boxplot of the accuracy for each model. The dots indicate the accuracy of each session and are visualized in chronological order (as in Figure 5). Linear regression lines indicate effects of time on the accuracy. Supplementary Table 2 lists the results of the significance tests. Significant correlation coefficients were obtained for the single session model (*r* = 0.52) and combined models without (*r* = 0.46) and with (*r* = 0.53) adaptation. The two-sample *t*-tests, comparing the first eight and last eight sessions, did not reveal significant differences.

The results for significant differences between the model accuracies are summarized in Figure 6A and listed in Supplementary Table 3. Comparing the transfer models, the feature normalization had a significant positive effect on the mean classification accuracy (*t* = 3.35). For the combined models, the effect did not turn significant (*t* = 0.95). There was no significant difference between the single session model and either transfer model. The combined models (with and without normalization) achieved significantly higher accuracies than the single session (*t* > 5.28) and transfer models (*t* > 6.63).

The transfer model with normalization corresponded to the pre-trained model that was used to provide feedback during the calibration runs (Figure 1B). Accordingly, the model’s average accuracy of 46 ± 4% (mean ± std) is comparable to the average online accuracy during the calibration runs of 44 ± 6% (mean ± std).

The confusion matrices of the combined model with feature normalization are summarized in Figure 6B. The feet class had the highest within-class accuracy, with 0.70 in the first eight sessions and 0.83 in the last eight sessions. The confusion among the other three classes was considerably larger, resulting in lower within-class accuracies. However, with prolonged training their within-class accuracies increased as well.

### Intra-Session Adaptation

**FIGURE 7:**
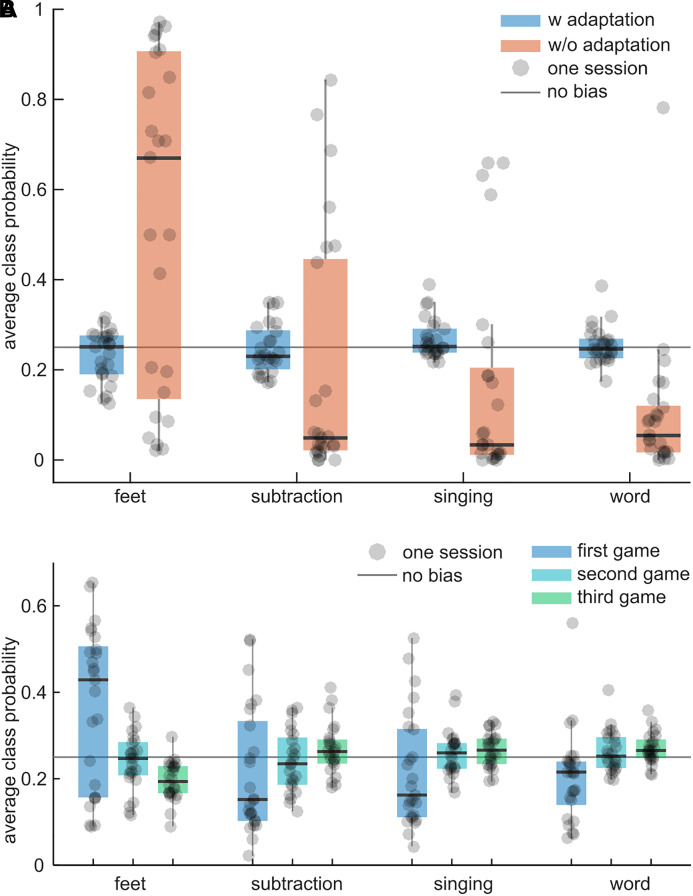
Intra-session adaptation vs. no adaptation. **(A)** Boxplots summarize the average class probability (feet, subtraction, singing, word; from left to right) during the games of an adaptive classifier (blue) and a non-adaptive classifier (orange). Each dot corresponds to one session and summarizes the average class probability within the session’s games. The solid gray line indicates the ideal average class probability (each class is equally probable). The larger the distance to this line, the larger the bias of a classifier toward one class. **(B)** As in **(A)** for the adaptive model and the first three games per session. Boxplots summarize the average class probabilities for the first, second, and third game.

To test whether the adaptive intra-session feature normalization technique reduces potential biases to specific classes during the BrainDriver games, the average difference between the class probabilities and 25% was compared. Ideally, the difference should be 0% because the classes’ duration and occurrence were approximately balanced during a game. Figure 7A illustrates the results for adaptive and non-adaptive models. Across sessions, the average class probabilities of the adaptive model were close to the ideal value of 25% and exhibited little variance, suggesting little to no biases for each session. The non-adaptive model exhibited session-specific biases with a systematic bias toward the feet class and a large variability across sessions. The grand-average differences to 25% were 3.8 ± 3.2% (mean ± std) and 26.2 ± 13.7% for the adaptive and non-adaptive models, respectively. The difference in the mean bias was significant (*t* = 14.55, *p* = 0.0001).

The average class probabilities of the adaptive classifier for the first three games are depicted in Figure 7B. In the first game, the adaptive classifier exhibited large biases toward feet but in subsequent games the biases became smaller and the mean probability was closer to 25%. This indicates that the adaptive normalization technique could compensate for distribution shifts between the calibration paradigm and the games within approximately one game run.

## Discussion

In this single case study, we applied inter-session transfer learning and unsupervised adaptation techniques to fit a 4-class BCI based on mental imagery to a user with tetraplegia with the goal of participating in a competition called BCI Race (Riener, 2016; Novak et al., 2017). We slowly adapted the BCI to the user-specific patterns by fitting the model parameters to calibration data of the current and five previous sessions. Within 14 months and 26 sessions, the system, comprising the tetraplegic user, the pilot, and the BCI, improved the classification accuracy from 46% in the first eight sessions to 53% in the last eight sessions. At the same time, the distances between the patterns significantly increased while the game runtimes significantly declined from 255 ± 23 s (mean ± std) to 225 ± 22 s. In addition to a training effect across sessions, we observed that adapting the normalization parameters of the log band power features within a training session mitigated intra-session non-stationarities. The unsupervised adaptation process was able to track shifts in the feature distribution, counteracting biases of the classification model during the BrainDriver game.

With training, the pilot was able to significantly improve the discriminability of the class specific patterns. Specifically, we observed stable patterns for the feet and subtraction tasks, while the patterns for singing and word were less consistent (Figure 4). Within the 14 months, the pilot could significantly increase the ratio of the between to within class pattern distances for the (feet, subtraction), (feet, word), and (subtraction, word) tuples (Supplementary Figure 2 and Supplementary Table 1). This matches with less confusion of the classifier for these tuples. For example, given feet was the target class, the misclassifications to the subtraction and word classes reduced by 5% and 6% from the first eight to the last eight sessions (Figure 6B). However, the moderate accuracies at the single trial level (Figure 6A) together with considerable overlaps of the class distributions (Figure 4B and Supplementary Figure 1) indicate large non-task related, trial-by-trial variability. Still, the significant accuracies of the transfer models (Figure 6A) suggest the presence of stable patterns. For example, the transfer model, fitted to the previous five sessions, could be applied to data of the new session without retraining, and achieve similar classification accuracies as a model that was fit to session specific data only.

Observing similar accuracies for session-specific and transfer models is surprising, since higher accuracies are typically reported for session-specific models than for transferred models (Lotte et al., 2018). This discrepancy can be partially explained by the curse of dimensionality problem. In our case, only three calibration runs with 30 trials per class were available per session, yielding a low trial to feature ratio for the single session models and leading, in turn, to overfitting. Among the tested models, the combined model, which used data of the current and previous sessions, achieved the highest classification accuracy (Figure 6A). This observation is in accordance with previous works (Lotte et al., 2018). Interestingly, feature normalization had no effect on the accuracy of the combined model, while it had a weak significant effect on the transfer model (Figure 6A). This result suggests that there were no major inter-session non-stationarity effects in the six consecutive sessions considered by the transfer and combined models. Indeed, looking at feature distributions without normalization (Supplementary Figure 3A), we observed rather small distances and class-specific clustering in sessions belonging to the same group (first, middle, last). Across groups, there was a gradient; observations of the first sessions were overall closer to the ones of the middle sessions than to the ones of the last sessions. This suggests that the long-term non-stationarities in the calibration data, which occurred over the course of the study (14 months), were higher than the non-stationarities between consecutive sessions. Normalizing the observations per session was effective in reducing these long-term non-stationarities (Supplementary Figure 3A).

While session-wise feature normalization had no significant effect on the classification accuracy of the combined model in the calibration data (Figure 6), unsupervised intra-session adaptation of the normalization parameters resulted in a significant effect on the class probabilities during games (Figure 7). Without adaptation, the calibrated model would have exhibited session-specific biases, suggesting the presence of shifts in the feature distribution when switching from the calibration paradigm to the BrainDriver game. Indeed, 2D visualizations of the calibration and game data feature distribution confirm the presence of a distribution shift (Supplementary Figure 5). Combining our observations, we can conclude that the switching of applications (calibration to game) introduced larger shifts in the subspaces of the feature space that are relevant for the sLDA classifier than across-session shifts (calibration to calibration). A model that adapted the normalization parameters was able to track changes in the overall feature distribution and reduce the biases within one game (Figure 7B).

Several approaches have been proposed to mitigate changes in the feature distribution (Vidaurre et al., 2011a; Samek et al., 2014; Morioka et al., 2015). Our results suggest that the unsupervised adaptation approach, originally presented in Vidaurre et al. (2011a), is suitable in this context. To be successful in compensating shifts in the feature distribution and maintaining class separability, this approach requires the data within the adaptation window to be balanced across classes. Here, the adaptation window corresponded to the approximate duration of a game, suggesting that the assumption is fulfilled. However, the game dynamics also depend on the pilot’s input. In our case, the pilot could trigger the different commands with varying degrees of accuracy (Figure 6B). Since the BCI could discriminate the feet class from the others with higher accuracy, he passed the associated sections faster, resulting in the feet class being the target class for shorter periods during a game. This becomes apparent, after the adaptation approach could compensate for the distribution shift between the calibration and game runs. For example, in the 3rd game run in Figure 7B, the probability of the feet class was on average 5% lower than the other classes’ ones.

Over the duration of the training period, we observed a significant decrease in the average runtimes (Figure 5). This is especially apparent in the periods leading up to the CYBATHLON BCI Series 2019, and the CYBATHLON 2020 Global Edition, respectively, where the density of training sessions was high, but even after training breaks of several weeks, performance held steady or slightly improved. The significant downwards trend in the game runtimes coincided with significant upwards trends in the calibration data metrics, specifically, in the pattern distance ratios (Supplementary Figure 2) and the classification accuracy (Figure 6A) of the combined with normalization model, which was used in the BCI during the games. Tests whether the effects were correlated (Supplementary Figure 4) revealed a significant correlation (*r* = 0.61, *p* = 0.0011) between the average pattern distance ratio and the classification accuracy. This result contrasts with findings from other studies with end-users, which observed that an increase in classification accuracy does not necessarily correlate with an enhancement in the separability of brain patterns (Leeb et al., 2015; Perdikis et al., 2016). Still, in our study neither the correlation between the runtimes and the classifier accuracy (*r* = −0.31, *p* = 0.1260) nor the correlation between the runtimes and the average distance ratio (*r* = −0.29, *p* = 1544) were significant. This means that a high performance in the calibration runs of a session was not significantly coupled with low runtimes during game runs. However, *p*-values close to 0.1 indicate that the variability in the data was too high to identify potential moderate to weak effects. The low correlations suggest that the runtime improvements were likely also driven by other factors. We speculate that the observed decrease of the runtimes could be linked to the pilot slowly learning to better time the triggering of the commands, rather than to an enhancement of the brain patterns *per se*.

Interestingly, in 2019, the pilot displayed some of his best runtimes in front of an audience. In the BCI Series 2019, he achieved a personal best, which remained unbroken for several months. Furthermore, in November 2019, he considerably outperformed his previous training average in a public demo performance, after a training break of over 2 months. These data points, in conjunction with our subjective impressions of how external factors seemed to influence his performance in training sessions, would support the hypothesis that our pilot tends to thrive in competitive settings. As a result of restrictions owed to the COVID-19 pandemic, we have not had further opportunities to put it to the test. The setting in the CYBATHLON 2020 Global Edition, while it was technically a competitive context, was not comparable in terms of actual circumstances. Due to COVID-19, the competition was modified to a decentralized format, where all races were individually recorded at the location of the team in remote correspondence with CYBATHLON headquarters prior to the broadcast day, and produced into a pseudo-live stream. As a result, the pilots had no real-time competition on the race track. In our specific case, the races also had to take place without an audience, and with only a small number of team members present, since our country was in a soft lockdown at the time.

We believe that these circumstances could partially explain why the official races in the CYBATHLON 2020 Global Edition did not rank among our pilot’s better training races in the preceding months. Still, our officially recorded race time of 223 s was within the range of race times that we observed in the last eight training sessions (225 ± 22 s). The time sufficed to achieve the fifth rank in the CYBATHLON 2020 Global Edition, with a relatively small margin of 2 and 10 s to the fourth and third place. The margin to the first (57 s) and second team (51 s) was considerably higher.

The fact that the winning team used two distinct motor imagery classes to implement the four commands (Perdikis et al., 2018) suggests that it is more efficient to detect two classes with a higher accuracy than four classes with low to moderate accuracy. As in Perdikis et al. (2018), the two classes could be mapped to two active commands, while the pilot could trigger the third command via a temporal coding. For example, the pilot could perform one mental task until the command is recognized. This will result in at least one false positive. However, this feedback informs the pilot to switch the mental task. If the BCI detects this switch within a certain time period, the system recognizes the temporal code and sends the third command to the game. The temporal coding strategy with limited mental tasks has proved useful in BCI research in other contexts (Müller-Putz et al., 2009, 2010). The confusion matrices of our four class system (Figure 6B) indicate that the feet and subtraction classes could have been detected with few misclassifications in a binary setting, rendering this strategy promising for future competitions.

Despite the promising results, our single case study suffers from a number of limitations. Foremost, we observed a large variation across runtimes of initially 23 s (std) that could not be reduced within the 14 months training period, confirming that reliability should be among the top priorities in BCI research (Lotte and Jeunet, 2015). Another limitation concerns the non-stationarities within a session. To avoid a situation like in the CYBATHLON 2016 (Statthaler et al., 2017), we tackled potential biases of the classification model, caused by within-session non-stationarities, 2-fold. First, we adapted the normalization parameters in an unsupervised fashion. Second, we applied an adaptive thresholding strategy that translated the classifier output into control commands. Since we applied both methods simultaneously, it is not possible to disentangle the individual effects on the performance in terms of runtime improvement. A systematic evaluation of the individual effects remains missing. Lastly, we decided to fix the features and mental tasks early after a few screening sessions so that we could collect a large dataset with the identical experimental protocol and BCI system. While this allowed us to identify training effects, we cannot rule out that other mental tasks or feature types, or advanced feature selection algorithms (Ang et al., 2008) might have led to higher classification accuracies and, in turn, to stronger training effects for our pilot.

## Conclusion

This longitudinal study has allowed us to gain valuable insights into long-term user training with a four class BCI based on mental imagery. Starting with weak patterns and low classification accuracies, the tetraplegic pilot was able to significantly improve his control skill over the course of the 14 months training period. At the end of the training period, the patterns of the classes feet and subtraction were separable with little confusion at the single-trial level. When additionally considering the classes singing and word, the confusion remained relatively high. Still, the classification accuracies and feature distributions showed that the separability among all four classes increased significantly with training. These improvements coincided with a significant reduction in the race times. The performance did not plateau toward the end of the training period, and importantly transferred to the CYBATHLON competitions, suggesting that the applied methods can facilitate user training and reduce inter- and intra-session non-stationarities. Future research is necessary to improve the reliability. Additional training sessions with the current methods are likely to result in gradual improvements.

## Data Availability Statement

The datasets for this article are not publicly available because it is a single case study and the name of the participant is known. Requests to access the datasets should be directed to GM-P, gernot.mueller@tugraz.at.

## Ethics Statement

Ethical review and approval was not required for the study on human participants in accordance with the local legislation and institutional requirements. The patients/participants provided their written informed consent to participate in this study. Written informed consent was obtained from the individual(s) for the publication of any potentially identifiable images or data included in this article.

## Author Contributions

RK and GM-P had the idea. LH, RK, CL-D, NS, and PT performed the data recording. LH, RK, CL-D, NS, PT, JU, and PRT performed the data analysis and made the figures. LH, RK, CL-D, and NS performed the paradigm design/implementation. LH, RK, CL-D, NS, PT, JU, PRT, and GM-P wrote the initial draft. LH, RK, CL-D, NS, PT, and GM-P wrote the manuscript. LH, RK, CL-D, NS, PT, PRT, and GM-P performed the proof-reading and editing. All authors contributed to the article and approved the submitted version.

## Conflict of Interest

The authors declare that the research was conducted in the absence of any commercial or financial relationships that could be construed as a potential conflict of interest.

## Footnotes

1 https://github.com/sccn/labstreaminglayer
