# Supplementary material for: Long-Term Mutual Training for the CYBATHLON BCI Race With a Tetraplegic Pilot: A Case Study on Inter-Session Transfer and Intra-Session Adaptation
Source: Front Hum Neurosci. 2021 Feb 26;15:635777. doi: 10.3389/fnhum.2021.635777 (PMC7952767; doi:10.3389/fnhum.2021.635777)
Supplement: Supplementary file 1 [file Table_1.pdf]

Long-term mutual training for the CYBATHLON BCI Race with a tetraplegic pilot: a case study on inter-session transfer and intra-session adaptation

Supplementary material

Authors

Lea Hehenberger[1,2,♪], Reinmar J. Kobler[1,2,3,♪], Catarina Lopes-Dias[1,2,♪], Nitikorn Srisrisawang[1,2,♬], Peter Tumfart[2,♬], John B. Uroko[2], Paul R. Torke[2] and Gernot R. Müller-Putz[1,2,4,𝄞]

[1] Institute of Neural Engineering; Graz University of Technology; Graz 8010, Styria; Austria
[2] Graz BCI Racing Team Mirage91; Graz University of Technology; Graz 8010, Styria; Austria
[3] Information Integration and Neuroscience Team; RIKEN Advanced Intelligence Project; Kyoto 619-0288; Japan
[4] BioTechMed Graz; Austria
Correspondence: gernot.mueller@tugraz.at

[♪] these authors contributed equally.
[♬] these authors contributed equally.

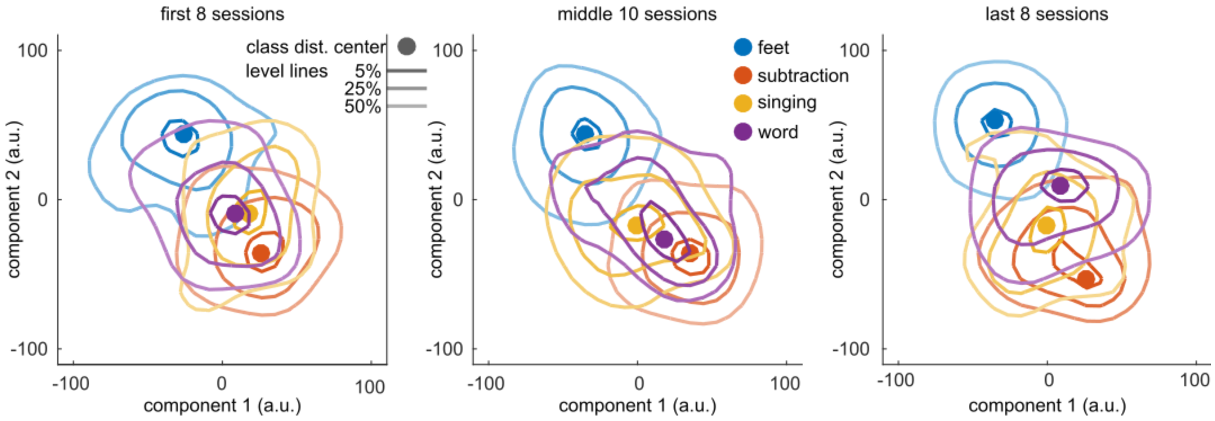

Supplementary Figure 1: Calibration data. Same plots as in Figure 4b but for multiple level lines that contain 5, 25 and 50% of the estimated probability densities. The dots indicate the distribution centers/modes.

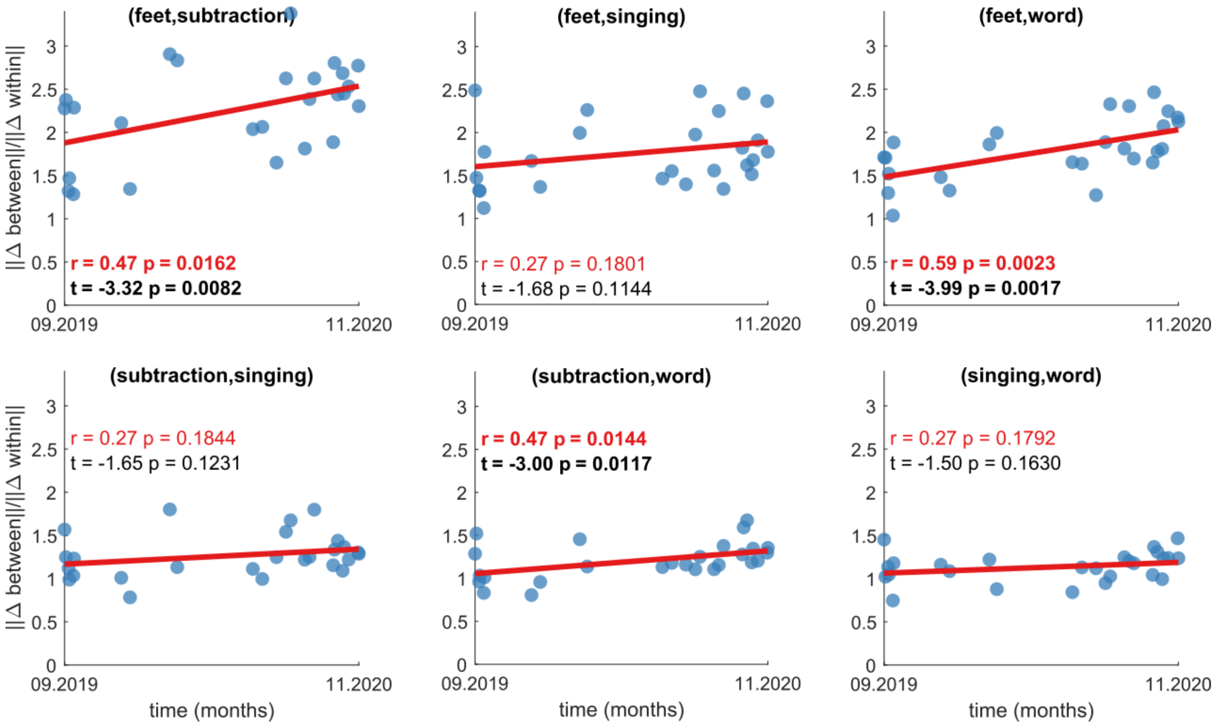

Supplementary Figure 2: Calibration data. Evolution of the between to within class distance ratios between binary class tuples. Each plot summarizes the results for one tuple. The dots indicate individual sessions. A regression line indicates linear trends. Correlation coefficients and t-statistics (between the first and last 8 sessions) are listed together with the associated p values. Significant effects are highlighted (bold fontweight, critical p value = 0.0162, corrected for 38 tests).

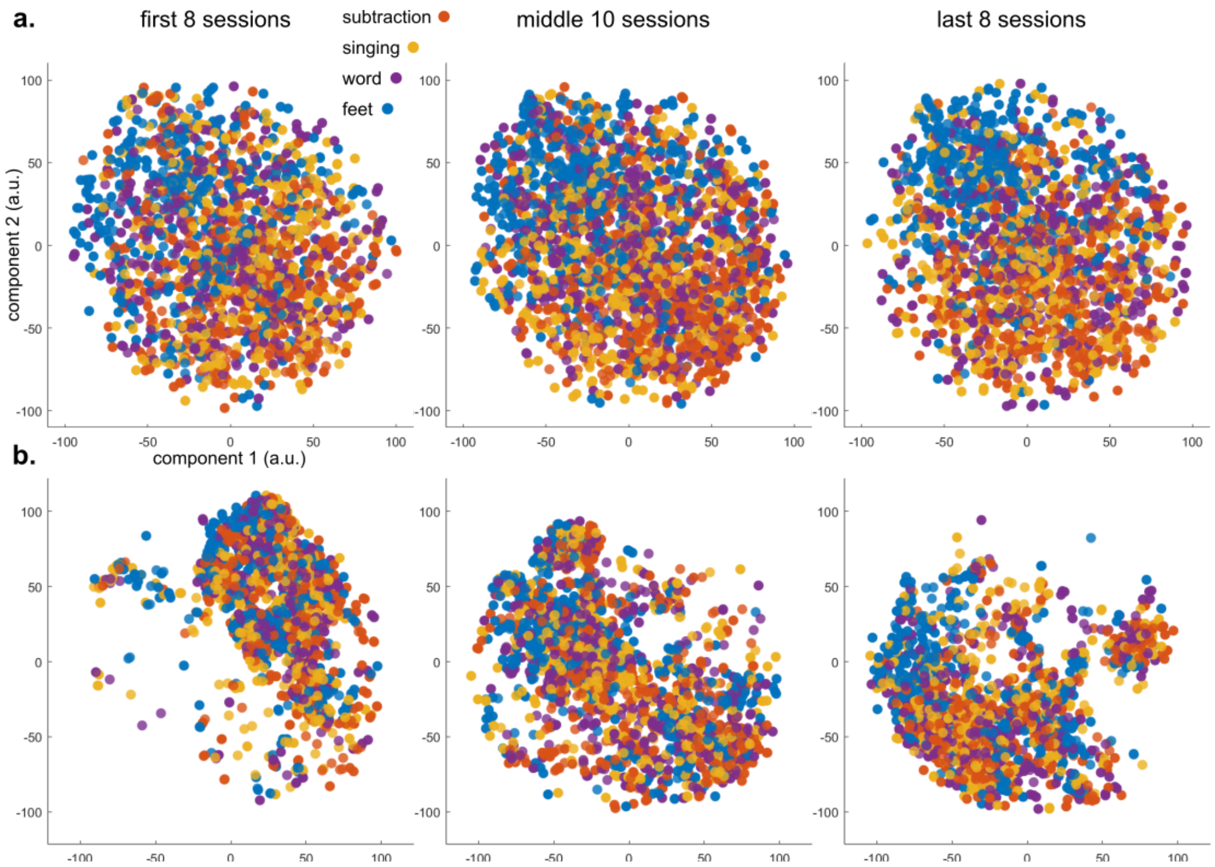

Supplementary Figure 3: Calibration data. Detailed visualization of the channel level log band power feature distribution. **a,** Relative power changes to the grand average session power, as in Figure 4b, Supplementary Figures 1 and 2. Each dot corresponds to one observation. The classes are color-coded. **b**, as in **a** without dividing the observations by the session-specific average.

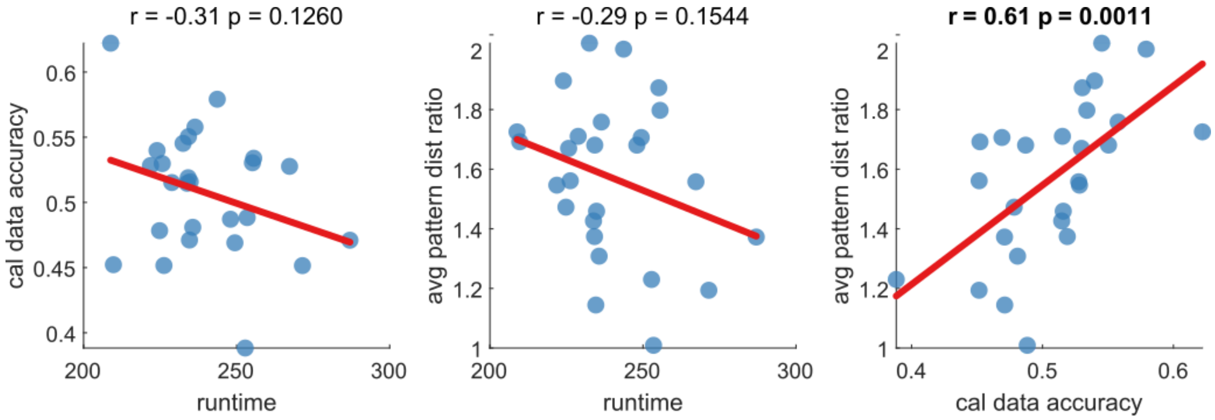

Supplementary Figure 4: Effects between the game runtimes, calibration data classification accuracy (combined with normalization model) and the average pattern distance ratio. Each dot corresponds to one session. The red line summarizes the linear dependency between the two metrics. Significant correlations coefficients and the associated p value are highlighted (bold fontweight, critical p value = 0.0162, corrected for 38 tests).

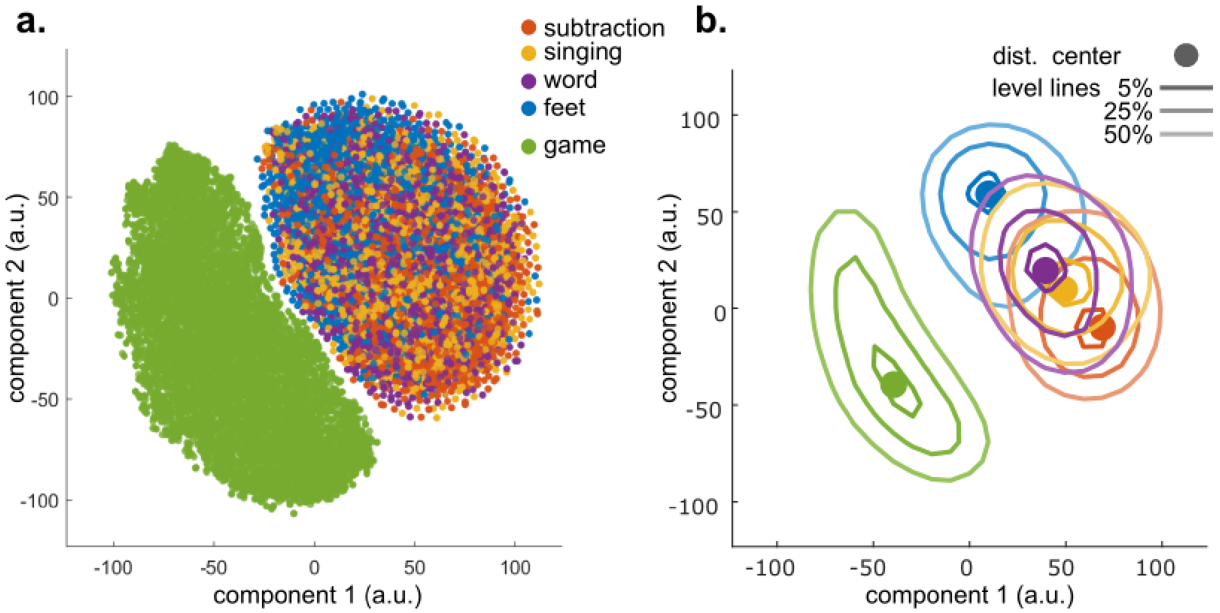

Supplementary Figure 5: Joint visualization of the log band power features for the calibration and game data of 25 sessions. **a,** Detailed visualization. Each dot corresponds to one observation. The calibration data observations are colored according to the associated class. The observations during the games are colored in green. All features were normalized with the session-specific average observation of the calibration data. **b,** estimated probability densities for the class-specific data in the calibration runs and the overall distribution in the game runs.

Supplementary Table 1: Calibration data. Patterns. Effect of training on the between/within class pattern distance ratio. For each binary tuple combining two classes, Pearson correlation coefficients (r) and t-statistics summarize trends across time and the difference between the first and last groups of 8 sessions. Significant differences are highlighted (bold fontweight, critical p value = 0.0162, corrected for 38 tests).

class1	class2	r	p	r_sig	t(first, last)	p(first, last)	t_sig
**feet**	**subtraction**	**0.47**	**0.0162**	TRUE	**3.32**	**0.0082**	TRUE
feet	singing	0.27	0.1801	FALSE	1.68	0.1144	FALSE
**feet**	**word**	**0.59**	**0.0023**	TRUE	**3.99**	**0.0017**	TRUE
subtraction	singing	0.27	0.1844	FALSE	1.65	0.1231	FALSE
**subtraction**	**word**	**0.47**	**0.0144**	TRUE	**3.00**	**0.0117**	TRUE
singing	word	0.27	0.1792	FALSE	1.50	0.1630	FALSE

Supplementary Table 2: Calibration data. Inter-session transfer accuracies. Effect of training on the calibration data accuracy. For each tested model, Pearson correlation coefficients (r) and t-statistics summarize trends across time and the difference between the first and last groups of 8 sessions. Significant differences are highlighted (bold fontweight, critical p value = 0.0162, corrected for 38 tests).

model	r	p	r_sig	t(first, last)	p(first, last)	t_sig
**single session**	**0.52**	**0.0070**	TRUE	2.21	0.0413	FALSE
transfer w/o norm	0.21	0.3158	FALSE	0.87	0.3942	FALSE
transfer w norm	0.26	0.1940	FALSE	1.06	0.3212	FALSE
**combined w/o norm**	**0.46**	**0.0148**	TRUE	1.82	0.0954	FALSE
**combined w norm**	**0.53**	**0.0052**	TRUE	2.40	0.0300	FALSE

Supplementary Table 3: Calibration data. Inter-session transfer accuracies. Effect of the model on the test set accuracies. For each binary tuple combining two models, two-sided, paired, permutation t-statistics were computed. Significant differences are highlighted (bold fontweight, critical p value = 0.0162, corrected for 38 tests).

model1	model2	t	p	significant
single session	transfer w/o norm	0.80	0.4378	FALSE
single session	transfer w norm	0.83	0.4138	FALSE
**single session**	**combined w/o norm**	**5.28**	**0.0001**	TRUE
**single session**	**combined w norm**	**5.82**	**0.0001**	TRUE
**transfer w/o norm**	**transfer w norm**	**3.35**	**0.0020**	TRUE
**transfer w/o norm**	**combined w/o norm**	**9.35**	**0.0001**	TRUE
**transfer w/o norm**	**combined w norm**	**8.63**	**0.0001**	TRUE
**transfer w norm**	**combined w/o norm**	**6.78**	**0.0001**	TRUE
**transfer w norm**	**combined w norm**	**6.63**	**0.0001**	TRUE
combined w/o norm	combined w norm	0.95	0.3469	FALSE
